# Editorial: Prodromal stage of neurodegenerative proteinopathies: from bench to bedside

**DOI:** 10.3389/fnins.2023.1295344

**Published:** 2023-09-27

**Authors:** Bin Xu, Seyed-Mohammad Fereshtehnejad, Yashar Zeighami

**Affiliations:** ^1^Biomanufacturing Research Institute and Technology Enterprise (BRITE), Department of Pharmaceutical Sciences, North Carolina Central University, Durham, NC, United States; ^2^Affiliated Faculty, Duke/UNC Alzheimer's Disease Research Center, Durham, NC, United States; ^3^Movement Disorders Clinic, Toronto Western Hospital, University of Toronto, Toronto, ON, Canada; ^4^Department of Neurobiology, Care Sciences and Society (NVS), Karolinska Institutet, Stockholm, Sweden; ^5^Department of Psychiatry, McGill University, Montreal, QC, Canada; ^6^Cerebral Imaging Center, Douglas Mental Health University Institute, Verdun, QC, Canada

**Keywords:** Alzheimer's disease, Parkinson's disease, prodromal stage, proteinopathy, early biomarkers, phenoconversion, mild cognitive impairment (MCI)

Neurodegenerative proteinopathies like Alzheimer's diseases (AD) and Parkinson's disease (PD) are preceded by a long period of prodromal stage. The pathologic changes of both AD and PD can start decades before the appearance of the debilitating cardinal symptoms, at which point they may be readily diagnosed. For instance, numerous prospective longitudinal studies have now proven that REM sleep behavior disorder, hyposmia, and dysautonomia start up to 20 years before any motor symptom manifests in synucleinopathy (Fereshtehnejad et al., 2019). Brain imaging, plasma, and cerebrospinal fluid (CSF) biomarkers, as well as subtle mild cognitive impairment (MCI) neurocognitive evaluation, have been used or discovered to assist in the timely and accurate screening of AD during early prodromal stages. Although very few disease-modifying agents have been discovered for AD, PD, or other neurodegenerative proteinopathies so far, enhancing our knowledge on various aspects of the prodromal stage is essential for timely diagnosis, designing clinical trials and eventual provision of neuroprotective therapy in the early stages of neurodegenerative proteinopathies.

It is speculated that one major reason for the failure of numerous clinical trials in neurodegenerative disorders is targeting the pathologic process in the later stage since by the time of diagnosis the majority of the neurons in the functional system of interest have already been irreversibly damaged. There is a recent shift, however, in the design of such clinical trials by recruiting participants within the prodromal stage of neurodegeneration hoping for a timely intervention. Furthermore, there is a dearth of knowledge on molecular pathogenesis, mechanisms and practical biomarkers to monitor disease trajectory during the prodromal stage, and/or accurately predict phenoconversion to a full-blown neurodegenerative disorder. Relentless basic science research is needed to understand the molecular interactions and pathways, to discover early disease biomarkers and potential neuroprotective agents targeting the responsible pathologic process to stop or slow down neurodegeneration during the prodromal stage when a functional amount of neurons and their connectors are still alive.

In the case of Alzheimer's disease (AD), neuropathological changes start one to two decades prior to the manifestation of cognitive decline and/or clinical diagnosis of dementia (Marrie et al., 2023). Early synaptic dysfunction and its resultant altered oscillatory activity in various neuromodulatory networks have been proposed as potential prodromal changes of Alzheimer's pathology (Pelucchi et al., 2022). In their study, van den Berg et al. at the University of Antwerp, Belgium demonstrated evidence of altered hippocampal network activity during the early stages of AD before the formation of plaques. They performed an open-field study in the TgF344-AD rat model with mutations in the amyloid precursor protein (APP) and presenilin-1 (PS1) at the age when only increased concentrations of soluble Aβ were present, but no Aβ-plaques had been formed yet. A decreased power of high theta oscillations and an increased sharp wave-ripple (SWR) in the CA1 layer of the hippocampus were detected, both of which suggest neuronal hyperexcitability occurring during presymptomatic stages of AD. These findings offer valuable insights into the malfunctioning of neural networks that occur prior to the onset of AD symptoms. As signs of prodromal hippocampal network impairment, alterations in theta-gamma coupling and sharp wave-ripple documented via electrophysiologic studies can be used to detect AD pathology during presymptomatic stages, with potential implications for future neuroprotective trials.

Similarly at the brain tissue level, locus coeruleus (LC) is well known to be associated with AD pathogenesis: There is up to 80% loss of LC neurons in AD (Bondareff et al., 1982). The integrity of the LC is associated with the main pathological and cognitive features of preclinical AD, suggesting that monitoring LC using non-invasive imaging methods might help to predict cognitive trajectories and AD pathology later in life (Jacobs et al., 2021). Li et al. at Soochow University, China measured the changes of the LC signals in early AD (MCI) and AD patients using neuromelanin-sensitive MRI technique, and analyzed its correlation with cognitive function. They found that the LC signal contrast ratios (LC-CRs) of AD patients but not of MCI subjects were significantly reduced from those of healthy controls. They further identified significant positive correlations between LC-CRs and the Mini-Mental State Examination (MMSE) sub item score in the AD group, and between LC-CRs and the Montreal Cognitive Assessment (MoCA) sub item score in the MCI group. These findings provide insights for future directions in mechanistic investigation and potential utility of LC-based imaging signals for translational AD diagnosis.

At the molecular level, aberrant phosphorylation of tau protein is a pathological hallmark of AD and several site-specific phosphorylation epitopes of the tau molecule including Ser396 have been proposed to be the early molecular events that play a pivotal role in AD pathogenesis (Wesseling et al., 2020; Wu et al., 2022). Yu et al. at Renmin Hospital of Wuhan University, China investigated the effects of a novel player, a fragment (amino acids 1–357) of γ-adducin (Xiong et al., 2021), a cytoskeletal protein, on tau phosphorylation and the kinases involved in this process. γ-adducin presents at spectrin-actin junctions in dendritic spines of hippocampal neurons and regulates synaptic plasticity, neurite growth, and degeneration. Using both *in vitro* assays and *in vivo* animal studies, they discovered that γ-adducin 1–357 fragment, but not the full-length γ-adducin, enhances tau phosphorylation at Ser396. The expression of γ-adducin 1–357 fragment leads to the activation of glycogen synthase kinase-3β. These findings identified a potential early event of dysregulation in the molecular pathways that lead to neurodegeneration and also a new target for mechanistic elucidation and for devising novel therapeutic strategies for AD treatment. As numerous tau phosphorylation epitopes are associated with the early stage in AD progression, significant work remain to be performed in this direction to gain insights into epitope-specific and/or stage-specific molecular pathogenesis mechanisms.

Similar to tau phosphorylation in AD, aberrant aggregation of α-synuclein proteins (called Lewy bodies) is one of the main hallmarks of PD (Bloem et al., 2021). While monogenic forms constitute a minority of PD cases, they provide invaluable information about the underlying pathophysiology of the disease (Ye et al., 2022). Mutations in SNCA, the gene encoding α-synuclein protein, have been identified as one of the causes of such monogenic PD cases. Morley et al. from the University of Edinburgh, used CRISPR/Cas9 technology to introduce G51D mutation into the endogenous rat SNCA gene. This was followed by ^18^F-DOPA positron emission tomography imaging at 5, 11, and 16 months, to assess the ratio between dopamine metabolites and dopamine itself. They discovered a significant increase in dopamine turnover in an asymmetric manner in the striatum of 16 months old SNCA^G51D^ rats, similar to observations in the PD cases. These results suggest that SNCA^G51D^ rats can be used as a new genetic model to study the dopaminergic denervation asymmetry observed in early stages of PD as well as the potential compensatory changes in dopaminergic system involved in prodromal PD.

Overall, this Research Topic highlights recent development and innovation in the field of mechanistic and translational investigation into the early stages of neurodegenerative diseases, including AD and PD. The Research Topic covers a range of original research articles in basic science, clinical, and translational studies. It describes new methods and technologies that may have translational values. It reports a novel molecular player that is a new neuronal target for mechanistic understanding in disease pathogenesis and for disease therapy. This Topic also characterizes a novel animal model that is capable to identify a highly relevant early disease phenotype. Our goal as editors was to provide a summarized overview representing the current state of the field and to identify future directions for research and development in this important area and we hope the readers will find interest in the included research articles.

## Author contributions

BX: Conceptualization, Data curation, Formal analysis, Investigation, Validation, Writing—original draft, Writing—review and editing. S-MF: Conceptualization, Data curation, Formal analysis, Investigation, Validation, Writing—original draft, Writing—review and editing. YZ: Data curation, Formal analysis, Investigation, Validation, Writing—original draft, Writing—review and editing, Conceptualization.

## References

[B1] Bloem B. R. Okun M. S. Klein C. (2021). Parkinson's disease. Lancet 397, 2284–2303. 10.1016/S0140-6736(21)00218-X 33848468

[B2] Bondareff W. Mountjoy C. Q. Roth M. (1982). Loss of neurons of origin of the adrenergic projection to cerebral cortex (nucleus locus ceruleus) in senile dementia. Neurology 32, 164–168. 10.1212/WNL.32.2.164 7198741

[B3] Fereshtehnejad S. M. Yao C. Pelletier A. Montplaisir J. Y. Gagnon J. F. Postuma R. B.. (2019). Evolution of prodromal Parkinson's disease and dementia with Lewy bodies: a prospective study. Brain 142, 2051–2067. 10.1093/brain/awz111 31111143

[B4] Jacobs H. I. L. Becker J. A. Kwong K. Engels-Domínguez N. Prokopiou P. C. Papp K. V.. (2021). *In vivo* and neuropathology data support locus coeruleus integrity as indicator of Alzheimer's disease pathology and cognitive decline. Sci. Transl. Med. 13, eabj2511. 10.1126/scitranslmed.abj2511 34550726 PMC8641759

[B5] Marrie R. A. Maxwell C. J. Rotstein D. L. Tsai C. C. Tremlett H. (2023). Prodromes in demyelinating disorders, amyotrophic lateral sclerosis, Parkinson disease, and Alzheimer's dementia. Rev Neurol. S0035-3787(23)00983-9. 10.1016/j.neurol.2023.07.002 37567819

[B6] Pelucchi S. Gardoni F. Di Luca M. Marcello E. (2022). Synaptic dysfunction in early phases of Alzheimer's disease. Handb. Clin. Neurol. 184, 417–438. 10.1016/B978-0-12-819410-2.00022-9 35034752

[B7] Wesseling H. Mair W. Kumar M. Schlaffner C. N. Tang S. Beerepoot P.. (2020). Tau PTM profiles identify patient heterogeneity and stages of Alzheimer's disease. Cell 183, 1699–1713. 10.1016/j.cell.2020.10.029 33188775 PMC8168922

[B8] Wu L. Gilyazova N. Ervin J. F. Wang S. J. Xu B. (2022). Site-specific phospho-tau aggregation-based biomarker discovery for AD diagnosis and differentiation. ACS Chem. Neurosci. 13, 3281–3290. 10.1021/acschemneuro.2c00342 36350059 PMC12316044

[B9] Xiong M. Zou L. Meng L. Zhang X. Tian Y. Zhang G. A.. (2021). γ-adducin cleavage fragment induces neurite deficits and synaptic dysfunction in Alzheimer's disease. Prog. Neurobiol. 203, 102074. 10.1016/j.pneurobio.2021.102074 33992672

[B10] Ye H. Robak L. A. Yu M. Cykowski M. Shulman J. M. (2022). Genetics and pathogenesis of Parkinson's syndrome. Annu. Rev. Pathol. Mech. Dis. 18, 95–121. 10.1146/annurev-pathmechdis-031521-034145 36100231 PMC10290758

